# *Ex vivo *and *in vivo *generation of neutrophil extracellular traps by neutrophils from septic patients

**DOI:** 10.1186/cc14116

**Published:** 2015-03-16

**Authors:** N Takeyama, MH Huq, M Ando, T Gocho, MH Hashiba, H Miyabe, H Kano, A Tomino, M Tsuda, T Hattori, A Hirakawa

**Affiliations:** 1 Fujita Health University, Aichi, Japan

## Introduction

The primary aim of this study was to determine the differences in *ex vivo *generation of neutrophil extracellular traps (NETs) by neutrophils from septic and nonseptic patients. We further sought to examine plasma levels of cell-free DNA (cf-DNA) and histones to assess *in vivo *NET formation.

## Methods

We isolated neutrophils from consecutive patients with sepsis (*n *= 17) and without sepsis (*n *= 18) admitted to the ICU. Neutrophils were activated by incubation with phorbol myristate acetate to induce release of NETs and NET formation was assessed by measuring the extracellular DNA level. Immunolabeling and fluorescence imaging were also performed. Extracellular killing of bacteria by NETs was studied by co-culture of *Escherichia coli *and neutrophils in the presence of the phagocytosis inhibitor cytochalasin D. To assess *in vivo *NET formation, plasma levels of cf-DNA and histones were measured.

## Results

The condition of the nonseptic patients was significantly less severe than that of the septic patients. The SOFA score of septic patients and the nonseptic patients was 6 (3 to 18) and 2.5 (1 to 8), respectively (median (IQR), *P *= 0.02). The overall mortality rate was 29%. After stimulation with PMA, neutrophils isolated from septic patients released 4.08 ± 1.02% of their total DNA, whereas neutrophils from nonseptic patients released 29.06 ± 2.94% (*P *< 0.0001). Immunofluorescent staining of released DNA, elastase, and myeloperoxidase also revealed similar results. Neutrophils from nonseptic patients showed effective extracellular killing of *E. coli *through NETs, whereas neutrophils from septic patients did not (*P *< 0.001). Plasma levels of cf-DNA and histones were higher in septic patients than in nonseptic patients (*P *< 0.001).

## Conclusion

The increase of the immature PMN count and immature/ total PMN ratio confirmed recruitment of immature neutrophils from the bone marrow into the circulation. The *ex vivo *generation of NETs is downregulated in neutrophils isolated from patients with sepsis. However, it is unclear whether *in vivo *NET formation is also impaired during sepsis, so further investigation is necessary.

